# Supplementary figures and images for: P2RX7 signaling drives the differentiation of Th1 cells through metabolic reprogramming for aerobic glycolysis
Source: Front Immunol. 2023 Mar 13;14:1140426. doi: 10.3389/fimmu.2023.1140426 (PMC10040773; doi:10.3389/fimmu.2023.1140426)

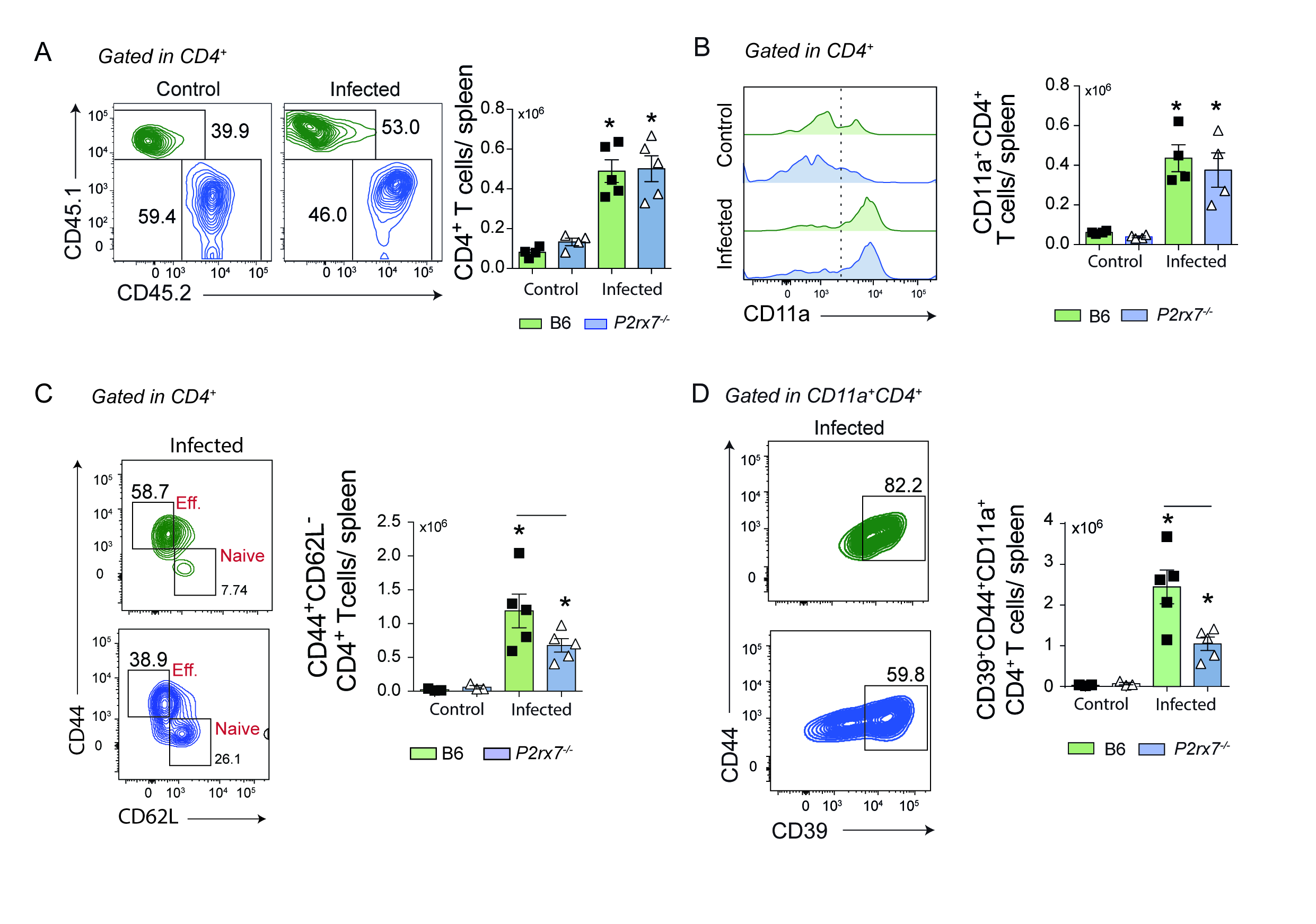

Supplement: Supplementary Figure 1 — Phenotypical analysis of co-transferred B6 and P2rx7-/- CD4+ T cells at early Plasmodium infection. Splenic naïve CD4+ T cells from B6 (CD45.1+) and P2rx7-/- (CD45.1+) mice were co-transferred into Cd4-/- mice that were infected with 1 × 10^6^ iRBCs. Splenic CD45.1+ and CD45.2+ CD4+ T cells were analyzed at day 6 of infection. Non-infected mice were used as controls. (A) Contour plots show CD45.1+ versus CD45.2+ expression in CD4+ T cells. Column bar graph shows CD45.1+ and CD45.2+ CD4+ T cell numbers per spleen. (B) Histogram shows CD11a expression in CD4+ T cells. Column bar graph shows CD11a+CD4+ T cell numbers per spleen. (C) Contour plots show CD44 versus CD62L expression in CD4+ T cells. Column bar graph shows CD44+CD62L-CD4+ (effector) and CD44-CD62L+CD4+ (naïve) T cell numbers per spleen. (D) Contour plots show CD44 versus CD39 expression in CD11a+CD4+ T cells. Column bar graph shows CD44+CD39+CD11a+CD4+ T cell numbers per spleen. Data are shown as the mean ± SD (n = 4-5) of one representative experiment out of three. Significant differences were observed for the (-) indicated groups and (*) between infected and control mice with p < 0.05, using Mann Whitney U test. [file Image_1.tif]

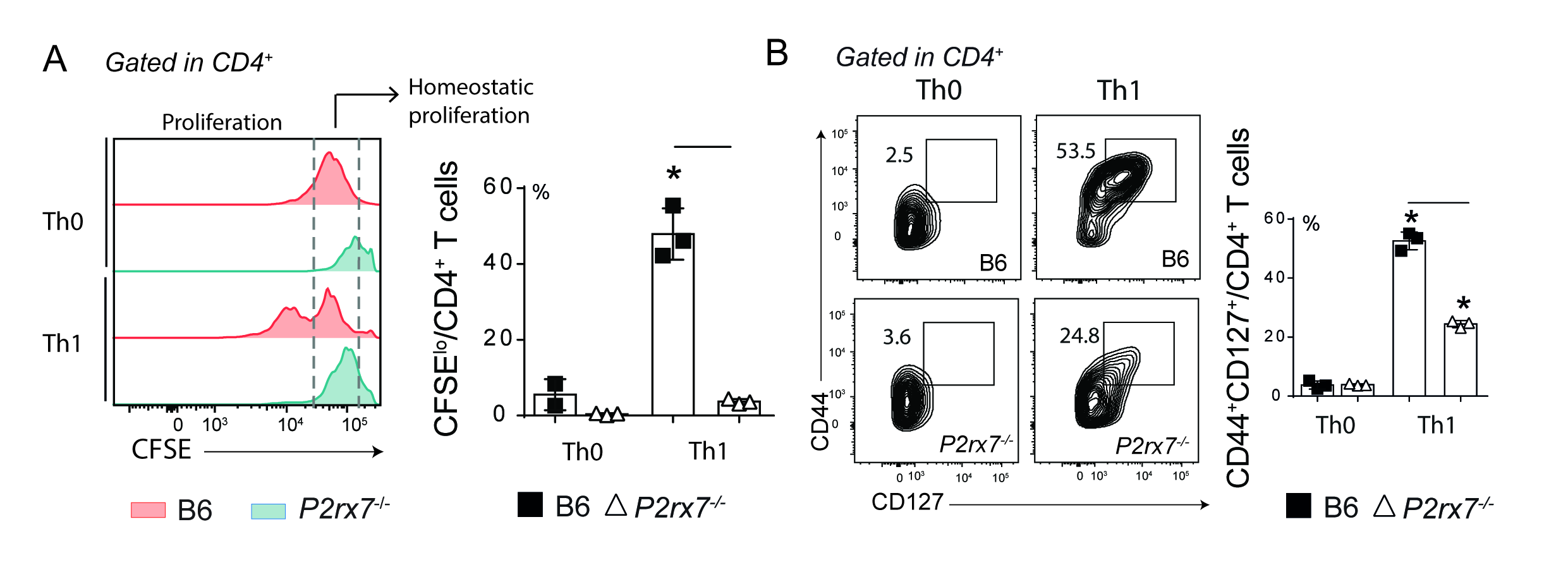

Supplement: Supplementary Figure 2 — CFSE staining and CD127 expression in Th1-conditionated CD4+ T cells that express or not P2RX7. Splenic naïve CD4+ T cells from B6 and P2rx7-/- female mice were activated under in vitro Th1 conditions. Th0 condition was used as control. (A) Histograms show CFSE staining CD4+ T cells. Column bar graph shows CSFEloCD4+ T cell frequencies. (B) Contour plots show CD44 versus CD127 expression in CD4+ T cells. Column bar graph shows CD44+CD127+ cell frequencies. Data are shown as the mean ± SD (n = 3) of one representative experiment out of three. Significant differences were observed for the (-) indicated groups with p < 0.05, using Mann Whitney U test. [file Image_2.tif]

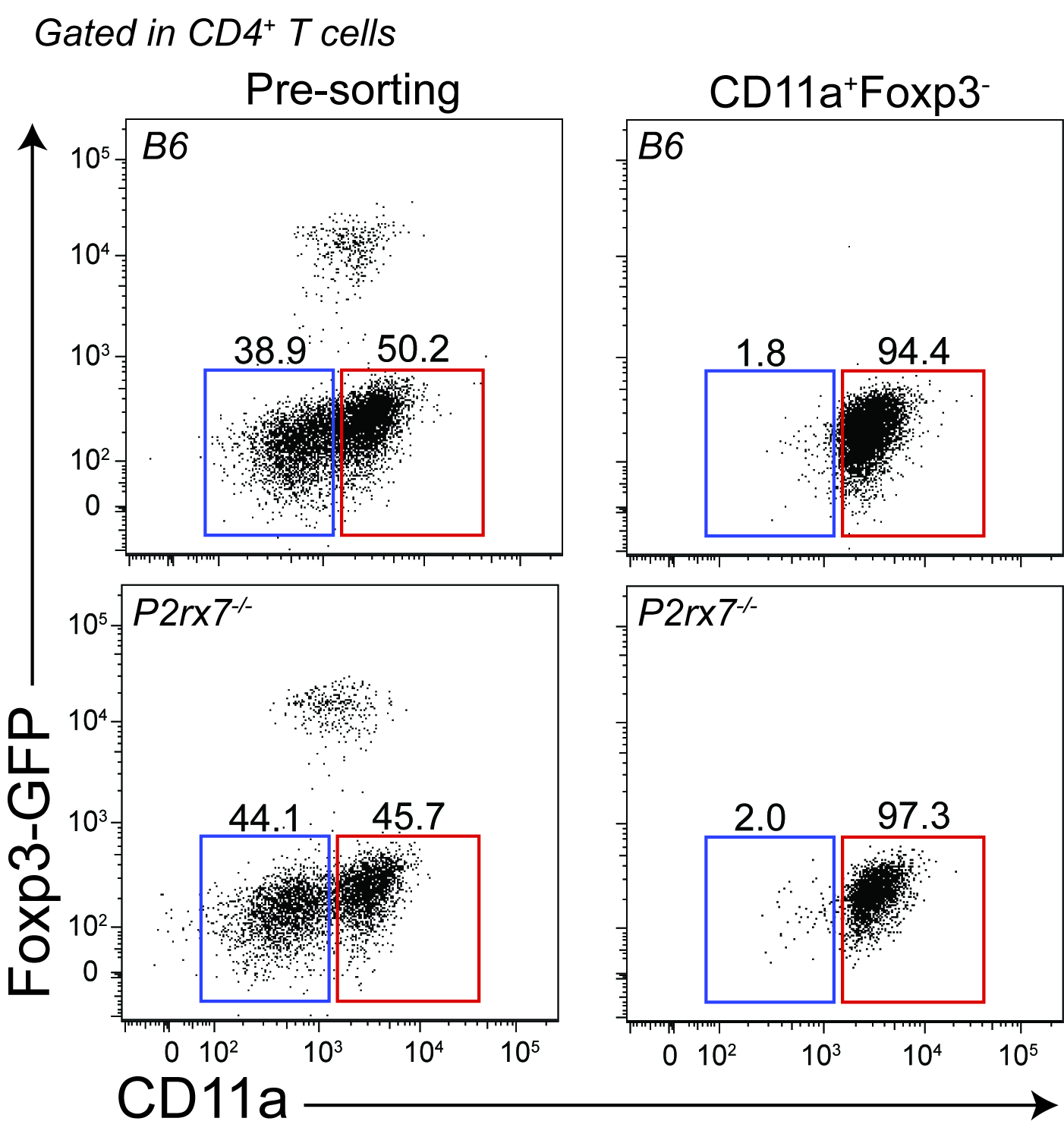

Supplement: Supplementary Figure S3 — Phenotypic analysis of sorted Foxp3-CD11a+CD4+ T cells. B6 and P2rx7-/- female mice were infected i.p. with 1 x 10^6^ iRBCs. At 6 days p.i., CD4+ T cells were purified using magnetic beads, and then Foxp3-CD11a+CD4+ T cells were sorted using a BD FACSARIA device. Data show one representative experiment out of six. [file Image_3.tif]
